# Burden of disease from shingles and post-herpetic neuralgia in the over 80 year olds in the UK

**DOI:** 10.1371/journal.pone.0229224

**Published:** 2020-02-25

**Authors:** Ian Matthews, Mai Duong, Victoria L. Parsons, Bayad Nozad, Nawab Qizilbash, Yash Patel, Boriana Guimicheva

**Affiliations:** 1 Merck Sharp & Dohme Limited, Hoddesdon, Hertfordshire, United Kingdom; 2 OXON Epidemiology, London, United Kingdom; 3 Department of Primary Care and Public Health, Imperial College London, London, United Kingdom; 4 London School of Hygiene & Tropical Medicine, London, United Kingdom; University of Illinois at Chicago, UNITED STATES

## Abstract

**Background:**

The current UK vaccination programme for herpes zoster (HZ) excludes people aged ≥80 years. This study aimed to quantify the number of individuals ≥80 years who missed HZ vaccination and the consequent epidemiological and economic burden of HZ and post-herpetic neuralgia (PHN).

**Methods:**

Immunocompetent individuals aged ≥80 years between 1^st^ September 2013 and 31^st^ December 2017 in the Clinical Practice Research Datalink were selected and linked to Hospital Episodes Statistics, where available. Rates of HZ and PHN and healthcare resource utilisation were investigated for the overall study population and by age group (80–84, 85–89, ≥90 years old) and the burden of HZ and PHN was projected to the UK population.

**Results:**

4,858 HZ episodes and 464 PHN cases were identified in 255,165 individuals over 576,421 person-years (PY). Rates of HZ and PHN were 8.43 (95% confidence interval [CI] 8.19–8.66) and 0.80 (0.73–0.87) per 1,000 PY respectively and lowest in those aged ≥90 (HZ rate 7.37/1,000 PY; PHN rate 0.56/1,000 PY). Within HZ episodes, 10.27% of GP visits, 5.82% of prescribed medications and 21.65% of hospitalisations were related to HZ/PHN. Median length of hospitalisation increased from 7.0 days for all-cause to 10.5 days for HZ/PHN related hospitalisations. Individuals ≥90 stayed in hospital a median of 3–4 days longer than younger groups. Approximately 2.23 million individuals in the UK missed HZ vaccination since 2013 (1.86 million had never been eligible and 365,000 lost eligibility for HZ vaccination), resulting in an estimated 43,149 HZ episodes.

**Conclusion:**

This study highlights the impact of the 80-year upper age limit policy on the health system. Our study estimates that 2.23 million individuals in the UK may have lost the opportunity to be vaccinated and that their burden of HZ and PHN remains high, especially among the very elderly.

## Introduction

Herpes zoster (HZ, shingles) is caused by the reactivation of the varicella zoster virus within the sensory ganglia [1, 2] and occurs most frequently in older adults. [3–5] The most common complication of HZ is post-herpetic neuralgia (PHN), a persistent neuropathic pain after the onset of the acute HZ episode. [6, 7] Older individuals are more likely to develop PHN and experience more severe acute pain. HZ and particularly PHN, have a major impact on patients’ quality of life. [8, 9]

The zoster-vaccine Live (ZVL), manufactured by Merck Sharp & Dohme UK, has been demonstrated to be effective in reducing the incidence of HZ and PHN in a randomised controlled trial. [10] In September 2013, the UK introduced a shingles national immunisation programme following the recommendation of the Joint Committee on Vaccination and Immunisation in 2010 [11]. ZVL was routinely offered to adults aged over 70 years and to those aged 78 to 79 years as part of a catch-up campaign. Although there have been some changes over subsequent years, the upper age limit of 80 years of age has remained consistent.

Three observational studies using primary care databases in the UK have demonstrated a good vaccine effectiveness (VE) of ZVL in both the routine cohort (aged 70 years) and the catch-up cohort (aged 78–79 years) with similar results reported for both cohorts, regardless of the age difference. [12–14] This finding is consistent with two cohort studies conducted in the US. [15, 16]

Public Health England reported a decrease in vaccine coverage over time for the catch-up cohort (from 57.8% in 2014–2015 to 46.2% in 2017–2018), suggesting a large proportion of those aged ≥80 who were previously eligible for vaccination had likely been missed. [17] Additionally, those who were over 80 at the start of the HZ vaccination programme in 2013 have never been eligible for the HZ vaccination. Using the Clinical Practice Research Datalink (CPRD) database linked with Hospital Episode Statistics (HES), this project aimed to assess the burden of HZ and PHN in unvaccinated immunocompetent individuals over 80 and to quantify the number of individuals who missed out on the HZ vaccination in the UK.

## Materials and methods

### Study design

This was a retrospective cohort study of individuals aged 80 years and older who were treated in routine UK care settings. Anonymised data from CPRD with linkage to HES data were used to investigate the burden of HZ and PHN. Data were available until 30^th^ June 2018 for CPRD and 31^st^ December 2017 for HES. CPRD is a primary care database of anonymised medical records from general practices (GPs) across the UK, collecting data from around 670 practices with over 11.3 million patients, and is broadly representative of the UK population in terms of age and sex. [18] Linkages to HES (hospitalisation data) are available for 75% of the English practices (equivalent to 58% of all CPRD practices). [18] The Read code system is used for coding diagnoses in CPRD while the International Classification of Diseases, Tenth Revision (ICD-10) system is used in HES data.

### Study population

Individuals aged ≥80 years (date of birth was set to 1st January as only year of birth is available in CPRD), registered with a GP in CPRD, with at least 12 months’ computerised data and had not transferred out of the practice or died before the start of the National Immunisation programme (1^st^ September 2013) were selected.

Index date was defined as the latest of the following dates: start of the vaccination programme, date turned 80 years old, CPRD entry date plus 12 months (to allow at least 12 months of historical data), or 12 months after the last HZ record before their 80^th^ birthday (to avoid including HZ episodes commencing before reaching the age limit).

Individuals diagnosed with primary or acquired immunodeficiency states and those prescribed immunosuppressive or immunomodulating therapy (see details S1 Appendix) prior to index date were excluded, as were those vaccinated against HZ. Additionally, for the PHN objectives, individuals with less than 3 months of follow-up after the first HZ record following index date were excluded to ensure sufficient data to identify PHN.

Individuals were followed up from index date until the earliest of: date of transfer out of the practice, death, last practice data collection date, end of CPRD data (30^th^ June 2018), date individuals became immunocompromised, or HZ vaccination date, whichever came first.

The study population were divided by their vaccine eligibility status into “never eligible” (i.e. aged ≥80 years when vaccination programme commenced) and a “lost eligibility” group (i.e. those who turned 80 during the vaccination programme).

### Outcomes

HZ was identified in both CPRD and HES through Read and ICD-10 codes. A HZ episode comprised of a single HZ record was defined as a period of 12 months following the HZ record. Where multiple HZ records existed, a gap of >12 months between two consecutive HZ records was required to separate two HZ episodes, as some patients may have more than one HZ episode.

PHN was identified within each HZ episode in two ways: 1) PHN diagnosis in CPRD or HES within 12 months of a HZ diagnosis; 2) PHN diagnosis in CPRD 3–12 months after the start of the HZ episode with at least one PHN medication prescribed on the same date, including oral/topical analgesics (co-codamol, co-dydramol, lidocaine or capsaicin), tricyclic antidepressants (amitriptyline, duloxetine, imipramine or nortriptyline) and anticonvulsants (gabapentin or pregabalin). Where multiple PHN records were identified within a HZ episode, only the first record was counted to avoid double counting.

Healthcare Resource Utilisation (HRU) was identified within each HZ episode, including GP visits and medications prescribed in primary care, and number and length of hospitalisations (defined as hospital stays of ≥24 hours). Each GP visit and hospitalisation was categorised as either HZ/PHN-related if there was a diagnosis of HZ/PHN on the same date of the GP visit or during the hospitalisation, or all-cause otherwise. Medications prescribed in primary care for HZ/PHN were antiviral medicines (aciclovir, valaciclovir and famciclovir) and PHN-related medicines (listed above) prescribed on the same day as an HZ or PHN diagnosis. HZ and PHN medications were chosen based on Clinical Knowledge Summaries of the National Institute for Health and Care Excellence. [19, 20] Length of hospital stay was calculated as the time from hospital admission to hospital discharge using HES admitted patient care data.

### Covariates

Variables to describe the study population included age, sex, history of HZ, region, with Townsend score of social deprivation and urban versus rural location reported where recorded in CPRD. In addition, comorbidities associated with increased incidence of HZ and conditions that could potentially be misclassified as PHN (e.g. trigeminal neuralgia, diabetic neuropathy and epilepsy) were reported. Individuals with missing data were excluded in relevant analyses. The study population was categorised into the three age groups: 80–84, 85–89 and ≥90 years based on their age during follow-up. Results were reported overall and for the three age groups, sex, social deprivation (Townsend Score 1 to 5, most to least affluent) and location (urban, rural).

### Statistical methods

Crude HZ rates per 1,000 PY with 95% Confidence Intervals (CI) were calculated as the number of HZ episodes divided by the number of PY at risk of HZ which was defined as the total follow-up time minus time with HZ. Crude PHN rates per 1,000 PY with 95% CI were calculated as the number of HZ episodes with PHN divided by the number of PY at risk of PHN which was defined as the total follow-up time minus the time between the first record of PHN within the HZ episode and the end of the HZ episode. Sensitivity analysis was conducted to investigate stability of HZ and PHN rates due to the birth date assumption, with date of birth changed to 1st July instead of 1st January.

Among patients with HZ, HRU outcomes were reported overall and for each age group by the total amount of resource used, proportion of HZ-PHN related resource and rate per PY with 95% CI. Length of hospital stays during HZ episodes was reported by mean (standard deviation [SD]) and median (interquartile range [IQR]).

Burden of disease from HZ and PHN was estimated by projecting the observed numbers of individuals in CPRD who missed out on HZ vaccination between 2013 and 2017, total number of HZ and PHN cases, rates of HZ and PHN and related HRU in CPRD to the UK population using the Office for National Statistics data. [21] To exclude immunocompromised and vaccinated individuals in the UK, the percentage decrease after applying exclusion criteria in the CPRD population was applied to the UK population. These percentages were calculated for each age group in CPRD, then applied to the UK population, to account for the difference of age between the CPRD and UK population.

### Ethics statement

The protocol for this research was approved by the Independent Scientific Advisory Committee (ISAC) of the Medicines and Healthcare Products Regulatory Agency (protocol number 18_320). The CPRD has been granted generic ethics approval for observational studies that make use of only anonymised data and linked anonymised NHS healthcare data (Multiple Research Ethics Committee ref. 05/MRE04/87).

## Results

**Fig 1:**
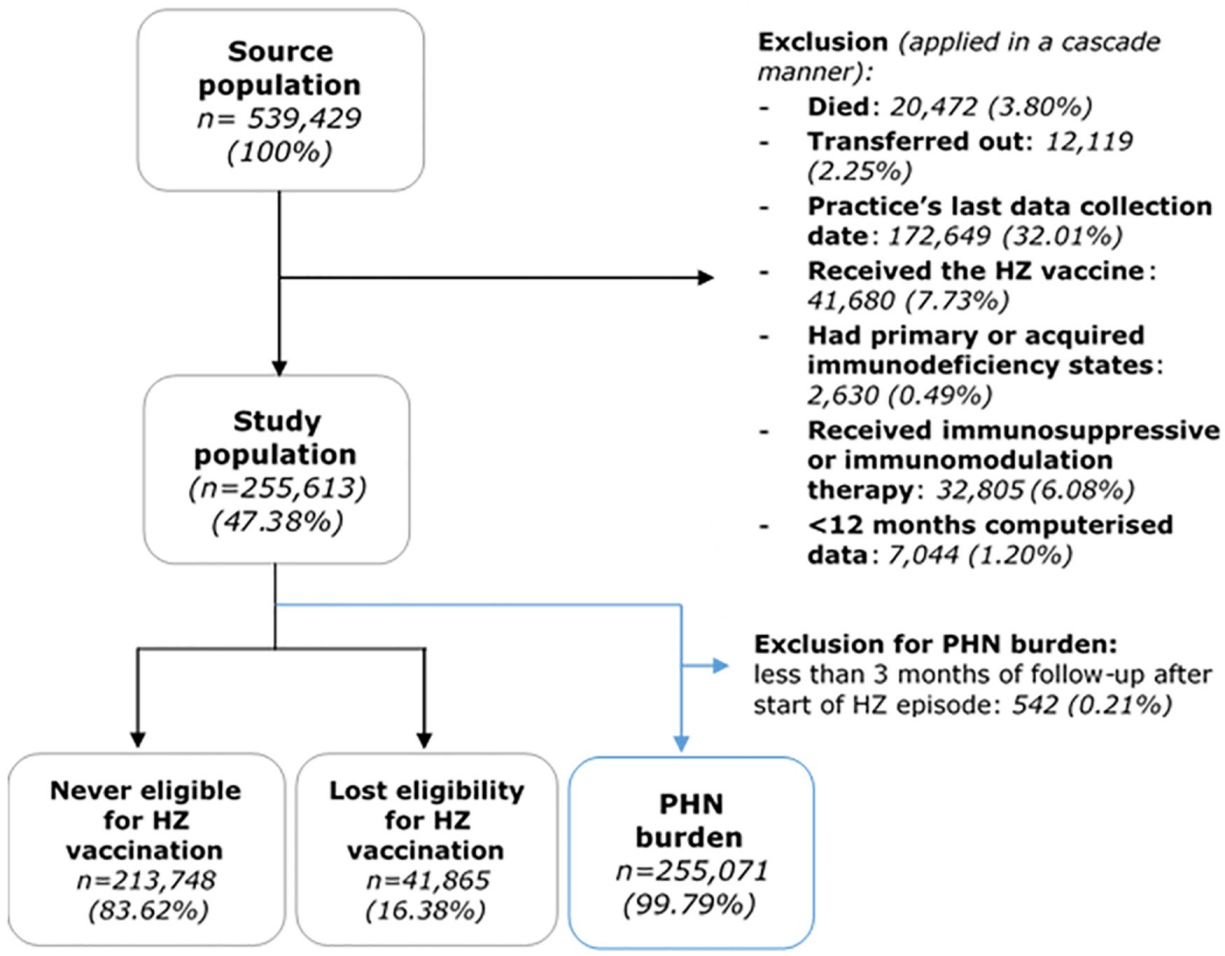
Study population selection process.

The final study population included 255,613 individuals, of these, 213,748 (83.62%) had never been eligible for HZ vaccination (aged ≥80 years at start of vaccine programme) and 41,865 (16.38%) had lost eligibility (turned 80 during the vaccination programme) (Fig 1). For PHN outcomes, a further 542 individuals with less than 3 months of follow-up after HZ diagnoses were excluded. HES data linkage was possible for 51.32% of the study population.

Individuals were followed up for a median of 1.96 years (IQR 0.86–3.56). Mean age at index date was 85.32 years (SD 4.90) with predominated females (61.59%) (Table 1). Most practices contributing to CPRD are based in England (16) and this was reflected in the data. Where practice postcode and social deprivation data were available, the majority (83.70%) lived in urban locations. The middle-to-high class proportion (Townsend score 1–3) was 71.72%, slightly higher than that of the general UK population. [22] 13.23% of the study population had a history of HZ at index date, 51.58% had at least one comorbidity that might be associated with increased risk of HZ and 6.22% had a history of conditions that could potentially be misclassified as PHN. As expected, those never eligible for HZ vaccination were older than those who had lost eligibility, however other clinical and sociodemographic characteristics were similar across the two groups (Table 1).

**Table 1:** Baseline characteristics of the study population and by subgroups of HZ vaccine eligibility.

	Overall study population		Never eligible for HZ vaccine (aged ≥80 years at start of study period)		Lost eligibility for HZ vaccine (turned 80 years during study period)	
	N = 255,613		N = 213,748		N = 41,865	
**Age at index date (years)**						
Mean (SD)	85.32	(4.90)	86.34	(4.72)	80.09	(0.43)
Median (IQR)	84.29	(81.67-88.67)	85.67	(82.67-88.89)	80.00	(80.00-80.00)
**Sex (n, %)**						
Male	98,191	38.41	79,997	37.43	18,194	43.46
Female	157,422	61.59	133,751	62.57	23,671	56.54
**Region (n, %)**						
England	180,946	70.79	153,681	71.90	27,265	65.13
Wales	37,646	14.73	29,674	13.88	7,972	19.04
Scotland	28,330	11.08	23,021	10.77	5,309	12.68
Northern Ireland	8,691	3.40	7,372	3.45	1,319	3.15
**Urban versus rural location**^¶^ **(n, %)**	145,108	100.00	123,299	100.00	21,809	100.00
Urban	121,460	83.70	102,833	83.40	18,627	85.41
Rural	23,648	16.30	20,466	16.60	3,182	14.59
**Social deprivation**^¶^ **(Townsend score) (n, %)**	140,058	100.00	118,922	100.00	21,136	100.00
1 (most affluent)	34,115	24.36	28,639	24.08	5,476	25.91
2	35,085	25.05	29,810	25.07	5,275	24.96
3	31,256	22.32	26,673	22.43	4,583	21.68
4	25,491	18.20	21,876	18.40	3,615	17.10
5 (least affluent)	14,111	10.08	11,924	10.03	2,187	10.35
**History of Herpes Zoster (n, %)**	33,821	13.23	28,647	13.40	5,174	12.36
**Any comorbidities associated with increased risk of HZ (n, %)**	131,844	51.58	111,613	52.22	20,231	48.32
Rheumatoid arthritis	5,338	2.09	4,410	2.06	928	2.22
Systemic lupus erythematosus	297	0.12	240	0.11	57	0.14
Inflammatory bowel disease	3,147	1.23	2,595	1.21	552	1.32
Diabetes mellitus	41,490	16.23	33,909	15.86	7,581	18.11
Chronic kidney disease	81,491	31.88	71,295	33.35	10,196	24.35
Chronic obstructive pulmonary disease	24,789	9.70	20,404	9.55	4,385	10.47
Asthma	33,238	13.00	27,439	12.84	5,799	13.85
**Any history of conditions that could potentially be misclassified as PHN (n, %)**	15,888	6.22	13,142	6.15	2,746	6.56
Trigeminal neuralgia	3,010	1.18	2,531	1.18	479	1.14
Diabetic neuropathy	8,283	3.24	6,856	3.21	1,427	3.41
Epilepsy	4,955	1.94	4,038	1.89	917	2.19
Any of the above	15,888	6.22	13,142	6.15	2,746	6.56

^¶^ Where recorded in CPRD

### HZ rates

**Fig 2:**
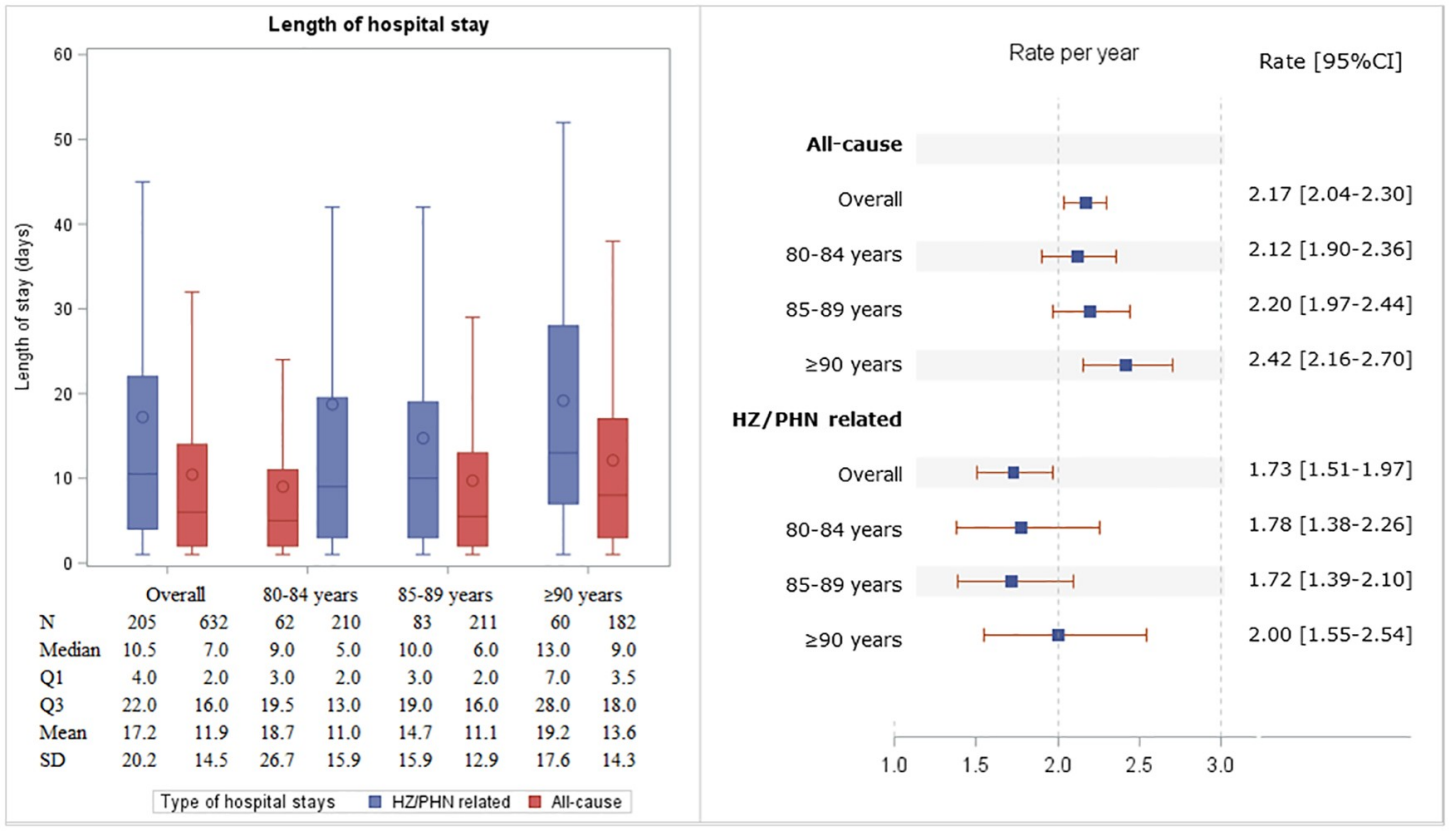
Rates of HZ and PHN in overall study population and for each stratification.

In total, 4,858 HZ episodes were identified over the study period (576,421 PY at risk), of which only 26 episodes (0.54%) were recurrent. 4,380 (90.16%) episodes were identified in individuals never eligible for HZ vaccine (aged ≥80 years at start of the vaccination programme) and 478 (9.84%) episodes were identified in individuals who lost eligibility (turned 80 during the vaccination programme). Overall rate of HZ was 8.43 (95% CI 8.19–8.66) episodes per 1,000 PY, with higher rates observed in those never eligible for HZ vaccination compared to those who lost eligibility (8.53 [8.27–8.78] vs. 7.62 [6.94–8.30] episodes per 1,000 PY) (Fig 2).

When stratified by age-group, HZ rates were similar between the 80–84 and 85–89 year old groups (8.85 [95% CI 8.46–9.23] and 8.64 [8.25–9.04] episodes per 1,000 PY respectively), however a lower rate was observed amongst those aged ≥90 years (7.37 [6.91–7.83] episodes per 1,000 PY) (Fig 2). HZ rate was higher in those with HES linkage compared to those without (9.05 [95% CI 8.68–9.42] vs. 7.94 [7.63–8.24] episodes per 1,000 PY), and among females compared to males (8.75 [8.44–9.05] vs. 7.91 [7.54–8.28] episodes per 1,000 PY). HZ rates tended to be slightly higher in more affluent groups and in people living in rural areas, though the confidence intervals overlapped (Fig 2).

### PHN rates

Over the study period, 464 PHN cases were identified over 578,800 PY at risk, corresponding to a rate of 0.80 (95% CI 0.73–0.87) cases per 1,000 PY. Those never eligible for HZ vaccination had a higher PHN rate compared to those who lost eligibility (0.82 [95% CI 0.74–0.90] vs. 0.65 [0.45–0.85] cases per 1,000 PY) (Fig 2). The pattern of PHN rates by age group, HES linkage, sex, social deprivation and location was similar to that of HZ (Fig 2).

In sensitivity analysis, no significant difference was observed in HZ rates when date of birth was set to 1^st^ July compared to the main analysis with date of birth set as 1^st^ January (S1 Table).

### Healthcare resource utilisation

HRU in primary care was quantified for 4,832 individuals with at least one HZ episode (Table 2). In total, 62,464 GP visits were identified during all HZ episodes, in which 6,418 (10.27%) were related to HZ/PHN. GP visit rate was 16 (95% CI 15.88–16.13) visits per PY for all-cause and 1.67 (1.63–1.71) visits per PY for HZ/PHN-related causes. Overall, 319,993 medications were prescribed over the course of all HZ episodes translating to a rate of 82.21 (95% CI 81.92–82.49) medications per PY, of which 18,621 (5.82%) were HZ/PHN medications (a rate of 5.55 [5.47–5.63] medications per PY) (Table 2).

**Table 2:** Healthcare resource utilisation within HZ episodes.

	Total amount of resource used	%	Number of person-years	Rate per person-year	[95%CI]
**General practice visits (N with HZ = 4,832)**					
All cause	62,464	100.00	3,903	16.00	[15.88–16.13]
Related to HZ/PHN	6,418	10.27	3,850	1.67	[1.63–1.71]
**Medications prescribed in primary care within HZ episodes (N = 4,832)**					
All cause	319,993	100.00	3,893	82.21	[81.92–82.49]
Related to HZ/PHN	18,621	5.82	3,353	5.55	[5.47–5.63]
**Hospitalisations (N with HZ and HES linkage = 2,287)**					
All cause	1,053	100.00	486	2.17	[2.04–2.30]
Related to HZ/PHN	228	21.65	132	1.73	[1.51–1.97]

**Fig 3:**
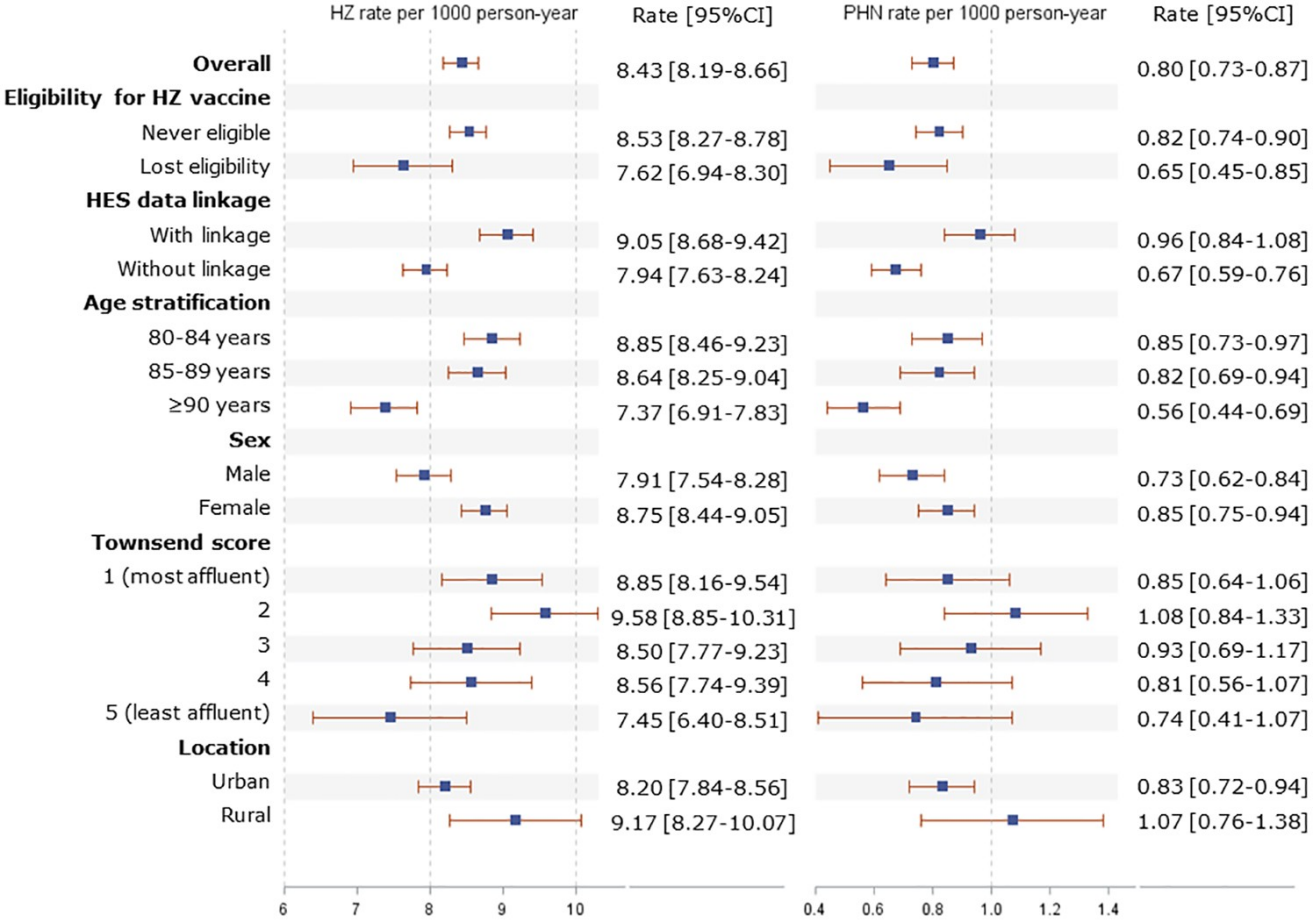
Rate of hospitalisation and length of hospital stays in individuals with 1 or more HZ episodes, overall and in each age stratification.

HRU in secondary care was estimated for 2,287 individuals with HES data linkage and at least one HZ episode (47.33% of all individuals with ≥1 HZ episode). Of these, 1,053 hospitalisations were identified in total, including 228 (21.65%) HZ/PHN-related. The rate of hospitalisation was 2.17 (95% CI 2.04–2.30) per PY for any cause and 1.73 (1.51–1.97) per PY for HZ/PHN-related causes. A total of 12,526 days in hospital were spent during all HZ episodes in which nearly a third (3,926 days; 31.34%) belonged to hospitalisations with a diagnosis of HZ/PHN (Table 2). Median length of stay was 3.50 days longer in HZ/PHN-related hospitalisations compared to those admitted for any cause (median 10.50 [IQR 4.00–22.00] vs. 7.00 [2.00–16.00] days) (Fig 3).

**Fig 4:**
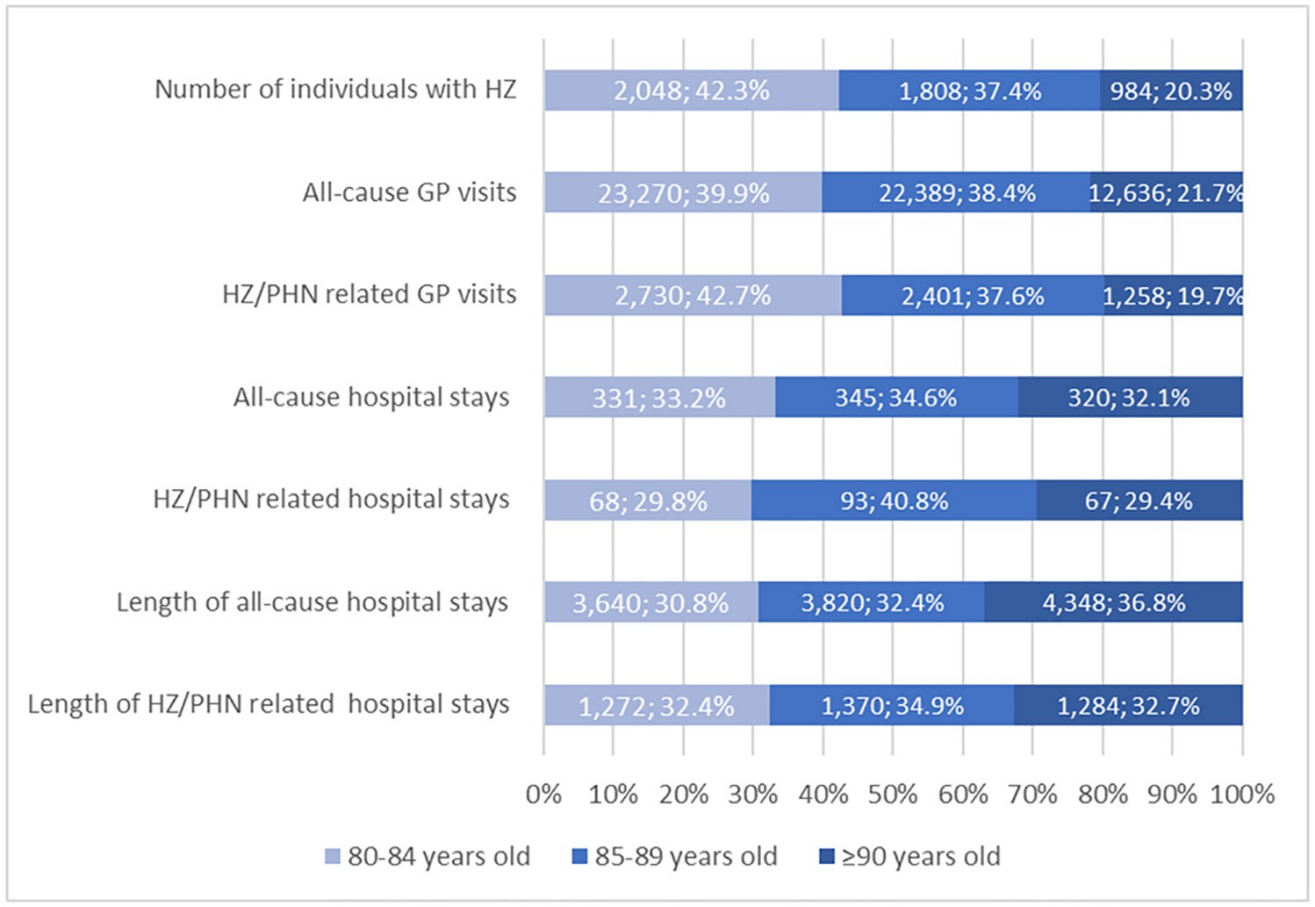
Number and proportion of healthcare resource utilisation by age in individuals with 1 or more HZ episodes.

GP visits, hospitalisations and length of hospital stay were described by age stratification (Fig 4). While the number of all-cause and HZ/PHN-related GP visits was proportional with the numbers of individuals with HZ in each age stratification (Fig 4), HRU in secondary care was notably higher in the older age group. Although those aged ≥90 years represented 20.33% of the study population with HZ, they accounted for nearly a third of the total number of hospitalisations. Additionally, median length of hospital stays in individuals aged ≥90 years was 3–4 days longer than that of the younger age groups for both all-cause and HZ/PHN related hospitalisations (Fig 4). Across three age groups, HZ/PHN related hospitalisations were a median 4 days longer compared to all-cause hospitalisations (Fig 4).

### Extrapolation to UK population

An estimated 2,229,831 individuals across the UK missed out on the shingles vaccine since the start of the national vaccination programme in 2013. This includes 1,864,623 individuals who were never eligible (i.e. aged ≥80 years at start of vaccination programme) and 365,208 individuals who lost eligibility due to the upper age limit (i.e. turned 80 during the vaccination programme). In the UK, a total of 43,149 HZ episodes were estimated to have occurred over the study period (20,686 episodes in 80–84 year olds, 15,882 in 85–89 year olds and 6,580 in ≥90 year olds), resulting in an age-adjusted HZ rate of 8.34 episodes per 1,000 PY (95% CI 8.26–8.42). For PHN, 4,046 cases were estimated across the UK during the study period (age-adjusted rate: 0.78 [0.76–0.80] per 1,000 PY). The projected HZ/PHN-related HRU in the UK within HZ episodes was 61,717 GP visits, 170,917 prescribed medications and 4,214 hospitalisations over the course of the study period.

## Discussion

This study is the first, to our knowledge, to quantify both the epidemiological and economic burden of HZ and PHN in individuals aged over 80 who missed out on HZ vaccination. The most important finding of this study is that the burden of HZ and PHN remained high in the ≥80-year olds and was especially notable in those who were never eligible for the vaccination, who represented 84% of the population missing out on HZ vaccination and accounted for 90% of all HZ episodes.

The HZ rate of 8.43 episodes per 1,000 PY in this study was within the range previously reported in the UK, [5, 12, 14, 23] and across countries in North America, Europe and Asia-Pacific (8–12 per 1,000 PY) in individuals over 80. [24] Our HZ rate was however slightly lower when compared to some non-UK studies, where incidence was estimated at 9.4 and 11.85 per 1,000 PY in individuals over 80 in the US and Germany, respectively. [15] The PHN rate of 0.80 per 1,000 PY in this study was higher than the previously reported in the UK for individuals aged 70–79 years of 0.53–0.70 per 1,000 PY [25], though this was somewhat expected since our study population was older. [14]

We observed a decrease in HZ and PHN rate in individuals over 90. To our knowledge, HZ incidence in the over 90 age group has not previously been reported in any studies to date. However, the decrease in HZ incidence in the oldest age groups has been previously reported in the UK (from 7.29 to 6.22 per 1,000 PY in 80–84 and ≥85 year olds, respectively) and Germany (from 9.7 to 9.4 per 1,000 PY among 75–79 and ≥80 year olds, respectively) [5, 25]. Potential explanations for the lower HZ and PHN rates are that a higher proportion of HZ and PHN cases may be identified in secondary care in the oldest age groups. Our data show that the rate of hospitalisation in ≥90 years old group was higher than in the two younger age groups. A decrease in prevalence of some chronic conditions from the age of 90 has also been reported for older people in England, which might also reflect a selective survival bias. [26] Furthermore, diagnostic difficulties are likely in the very elderly and could be compounded by the exponential increase in dementia in the older ages which may lead to underdiagnosis or misdiagnosis of HZ.

This study is the first to quantify the economic burden of HZ and PHN in those aged ≥80 years, which was considerable, particularly in the secondary care setting. The median length of hospital stay was 3.5 days longer in HZ/PNH-related stays compared to all-cause hospital stays. Moreover, the oldest age group (≥90 years old) experienced more and longer HZ/PHN-related hospital stays compared to younger age groups, indicating greater healthcare usage and potentially greater severity of illness in the eldest patients with HZ/PHN.

CPRD and similar primary care data have been used to investigate HZ and PHN previously. (16, 21, 22) One of the limitations of CPRD is that only year of birth is available for adults, therefore date of birth in this study was assumed to be the 1st of January. Whilst this approach may lead to underestimation of HZ/PHN incidence, no evidence of this was found in a sensitivity analysis (S1 Table).

Use of HES data permitted the identification of HZ and PHN cases in secondary care, however HES linkage was only available for practices in England (51% of the study population). Any HZ/PHN cases solely identified in secondary care for patients missing HES linkage would not be captured in this study, leading to potential underestimation of HZ and PHN rates. In addition, medications administered in hospital are not recorded in HES, therefore HZ/PHN medication use in hospital cannot be quantified. Finally, Townsend score for social deprivation and location (urban/rural) data were not available for the whole study population and individuals with missing data were not included in the relevant analyses.

In this study, HRU was estimated only during HZ episodes, therefore any under-diagnosis or under-recording of HZ and PHN would result in underestimation of HRU. Additionally, it might be difficult to quantify HRU solely caused by HZ and PHN since patients might have to visit GPs or be hospitalised for other chronic diseases regardless of the presence of HZ.

The benefit of HZ vaccine in individuals ≥80 years old would depend on the VE in this population. VE in individuals aged ≥80 years in the US has been shown to be similar to that of 70–79 year olds. [15, 16] However, as in younger age groups, waning effectiveness over a longer period should be considered while quantifying the benefit of the HZ vaccine in the ≥80 year old population, though life expectancy at this age is limited.

Finally, the HZ and PHN rates estimated in this study were not adjusted for any covariates as the objective of the study is to describe the burden in different sub-populations and any comparisons between different groups were based on crude rates and their CIs; no statistical comparisons were carried out. The extrapolated numbers in the UK were adjusted for age but not for other factors such as sex, geographic region and disease profile.

## Conclusion

This study is the first of its kind to quantify both the epidemiological and economic burden of HZ in unvaccinated individuals over 80 years old using real world data in the UK. The burden of HZ and PHN in unvaccinated individuals aged over 80 remains considerable, especially among the eldest age group. We estimated that 2.23 million individuals in the UK have missed out on the HZ vaccination because of the 80-year upper age limit. The study provides evidence for the UK to review the upper age limit policy.

## Supporting information

S1 Appendix Criteria used to identify immunocompromised individuals. (PDF)

S1 Table Sensitivity analysis of HZ rates in the overall study population and for each eligibility and age stratification with date of birth set to 1^st^ July instead of 1^st^ January, with % difference compared to the main analysis. (PDF)
