# Supplementary material for: Burden of disease from shingles and post-herpetic neuralgia in the over 80 year olds in the UK
Source: PLoS One. 2020 Feb 25;15(2):e0229224. doi: 10.1371/journal.pone.0229224 (PMC7041808; doi:10.1371/journal.pone.0229224)
Supplement: S1 Appendix — (PDF) [file pone.0229224.s001.pdf]

S1 Appendix. Criteria used to identify immunocompromised individuals

1. Individual has primary or acquired immunodeficiency states
    - Individual has primary or acquired immunodeficiency states within 24 months before index date, including:
        - Lymphoma
        - Myeloma
        - Other plasma cell dyscrasias
        - Leukaemia
        - Bone marrow transplant
        - Stem cell transplant
    - Individual has primary or acquired immunodeficiency states any time before index date, including:
        - Cellular immune deficiency
        - Solid organ transplants
        - HIV
2. Individual receives immunosuppressive or immunomodulation therapy
    - Individual receives immunosuppressive or immunomodulation therapy within 3 months before index date, including:
        - Azathioprine at a dose of ≥50mg/day
        - Methotrexate at dose of >25mg per week (>3.57 mg/day)
        - 6-mercaptopurine at a dose of ≥45mg/day
        - Other immunosuppressive agents at any dose
        - Other disease-modifying anti-rheumatic drugs e.g. mycophenolate, leflunomide at any dose
        - Injectable or oral steroids at >40 mg/day for >7 days, or at >20mg/ day for >14 days
    - Individual receives immunosuppressive or immunomodulation therapy within 12 months preceding index date, including:
        - Biological agents (e.g. anti-TNF therapy) at any dose
        - Cancer chemotherapy or radiotherapy at any dose
