# Supplementary material for: Burden of disease from shingles and post-herpetic neuralgia in the over 80 year olds in the UK
Source: PLoS One. 2020 Feb 25;15(2):e0229224. doi: 10.1371/journal.pone.0229224 (PMC7041808; doi:10.1371/journal.pone.0229224)
Supplement: S1 Table — (PDF) [file pone.0229224.s002.pdf]

S1 Table. Sensitivity analysis of HZ rates in the overall study population and for each eligibility and age stratification with date of birth set to 1st July instead of 1st January, with % difference compared to the main analysis

	N episodes	N person-years	Rate per 1,000 person-year [95%CI] (sensitivity analyses)	Rate per 1,000 person-year [95%CI] (main analyses)	% difference compared to main analysis
**Overall**	4,791	563,657	8.50 [8.26 – 8.74]	8.43 [8.19 – 8.66]	0.85%
**HZ vaccine eligibility**					
Never eligible	4,449	516,956	8.61 [8.35 – 8.86]	8.53 [8.27 – 8.78]	0.94%
Lost eligibility	342	46,701	7.32 [6.55 – 8.10]	7.62 [6.94 – 8.30]	-3.91%
**Age stratification**					
80-84 years	2,003	218,812	9.15 [8.75 – 9.55]	8.85 [8.46 – 9.23]	3.48%
85-89 years	1,905	211,885	8.99 [8.59 – 9.39]	8.64 [8.25 – 9.04]	4.01%
≥90 years	989	135,015	7.33 [6.87 – 7.78]	7.37 [6.91 – 7.83]	-0.61%
